# Ergot of Rye in Various Kinds of Hæmorrhages

**Published:** 1831

**Authors:** 


### ERGOT OF RYE IN VARIOUS KINDS OF HÆMORRHAGES.

IN a former paper we gave the experience of Messrs. Spairani and Pignacea respecting ergot of rye in several uterine affections—we now proceed to state their experience in hæmorrhagic affections of different organs.

### EPISTAXIS.

*Case* 1. A child, five years of age, was subject, for two years past, and without obvious cause, to epistaxis from the left nostril, which, however, was readily suppressed by the usual means. But sometimes the bleeding continued, more or less, for a considerable time after the principal hæmorrhage. Dr. S. prescribed eight pills, each containing four grains of the ergot of rye, and one to be taken every two hours. In a few hours the hæmorrhage ceased, and returned no more. The medicine was afterwards taken as a preservative.

*Case* 2. A young woman, aged 15 years, and who had not menstruated, was seized with gastric inflammatory fever on the 10th of August, 1829. On the 14th, in the evening, hæmorrhage took place from the left nostril, and went on to an alarming extent. This hæmorrhage was habitual; but had hitherto been easily restrained. This time it had continued 16 hours when the reporter arrived. A drachm of the ergot of rye was divided into six equal parts, one to be taken every ten minutes. The nostril was cleared of all clots of blood, and no mechanical means of arresting the hæmorrhage were used. The hæmorrhage continued during the exhibition of the medicine; and a second quantity was ordered. Immediately after the exhibition of the first dose from the second batch, the bleeding stopped. The ergot was continued through the day. During the succeeding two days some drops of blood oozed from the nostril, then stopped entirely. The fever ran its usual course—the patient recovered—and lost her habitual epistaxis.

### HÆMOPTYSIS.

*Case* 1. A female, aged 42 years, and who had ceased to menstruate, and who was subject to pulmonic affections, consulted the narrator in the Autumn of 1828, on account of a severe cough, accompanied by sanguineous expectoration, and sometimes pure blood. There was no fever; but the pulse was hard and full—the respiration short and embarrassed. Venesection and purgation were prescribed, with rigid diet. Next day the pulse was natural; but the discharge of blood from the lungs continued. Another bleeding was ordered, and a grain of digitalis every two hours. On the third day, the hæmoptysis continued, though the other symptoms were mitigated. A drachm of the ergot, divided into eight doses, was ordered to be taken within the 24 hours. After the fifth dose there was not a trace of blood in the expectoration. Another drachm, however, was administered, and no more hæmorrhage was seen.

*Case* 2. M. G. a young man, aged 21 years, was seized with a severe cough, accompanied by sanguinolent expectoration, in the Summer of 1828, in consequence of stripping off his clothes during a profuse perspiration. Fever and palpitation were added to the other symptoms. Venesection, quietude—diluents—digitalis, &c. relieved the cough, fever, and palpitation; but the hæmoptysis continued. A drachm of the ergot was therefore administered in 24 hours, and the hæmorrhage was completely arrested. In order to prevent a relapse, another drachm was given in the course of two days. Some months afterwards there was a return of hæmorrhage, though slight in degree; and the young man sent, of his own accord, for a drachm of the ergot, which stopped the discharge.

*Case* 3. Madam N. B. 72 years of age, had suffered of late years from severe catarrhal affections. In the month of July, 1828, she had a fall, and bruised the left side of the chest; which was followed by cough and spitting of blood. She disregarded the accident, and pursued her usual regimen. The reporter was called on the 20th day after the accident, and, considering the complaint as a topical affection, ordered some leeches to the side, and prescribed low regimen. A drachm of the ergot was ordered in the 24 hours; and as she was in the habit of drinking wine, the usual quantity was allowed, in order to ascertain more correctly the effects, if any, of the medicine. In the course of the next day, the blood disappeared from the expectoration, and was no more seen.

*Case* 4. A. M. a young girl, about the age of 12, had suffered for some time from severe catarrh. On the 26th January, 1829, she expectorated blood, and more or less of this fluid continued to be ejected by coughing. Half a drachm of the ergot was directed in the 24 hours. The sanguineous expectoration nearly ceased within that time; but the medicine was continued for four days, when it ceased entirely.

Hematuria.

*Case* 1. Mr. C. a gentleman, about 70 years of age, was attacked by ischuria, which resisted the usual remedies, and the catheter was introduced for 20 days in succession. At this time a considerable hæmorrhage took place from the urethra, which continued and became alarming. The ergot was prescribed, and after the first dose, there was no more blood passed from the urethra.

Several other cases are related, where the ergot succeeded in restraining hæmorrhages from the lungs, the womb, the bladder, and other parts; but we think the foregoing are sufficient to excite the attention of the profession to the remedy. There is this recommendation to the article, that there appears to be no possible *objection* to its exhibition. It is not an excitant or stimulant. If it possesses any properties, they are of the *specific* class—that is—they are not under the dominion of any general rules relating to the animal economy.—Transactions Medicales.

